# Effect of enhanced early life nutrition on the molecular regulation of anterior pituitary function in Holstein Friesian bull calves

**DOI:** 10.1038/s41598-025-04176-0

**Published:** 2025-07-01

**Authors:** K. Keogh, S. Coen, P. Lonergan, S. Fair, D. A. Kenny

**Affiliations:** 1 Animal and Bioscience Research Department, Teagasc, Animal & Grassland Research and Innovation Centre, Grange, Dunsany, Co. Meath Ireland; 2 School of Agriculture and Food Science, University College Dublin, Belfield, Dublin 4, Ireland; 3 Laboratory of Animal Reproduction, Department of Biological Sciences, University of Limerick, Limerick, Ireland

**Keywords:** RNAseq, Proteomics, Network analysis, MiR-205, Proteomics, Transcriptomics

## Abstract

Enhanced early-life nutrition is known to induce precocious reproductive development in the bull calf, mediated through gonadotropin releasing hormone (GnRH) stimulated gonadotropin pulsatility in the anterior pituitary gland. The objective of this study was to evaluate transcriptomic and proteomic responses within the anterior pituitary of Holstein–Friesian bull calves offered different planes of nutrition during early life. Bull calves were offered either a high (HI; n = 15) or moderate (MOD; n = 15) plane of nutrition between 2–12 weeks of age and subsequently euthanised at 12 weeks of age. The anterior pituitary tissue was harvested from all calves and miRNAseq, mRNAseq and proteomic analyses undertaken. High diet calves displayed greater growth rates compared to MOD calves (P < 0.001). Overall, 37 mRNAs and 5 miRNAs were differentially expressed between treatment groups (FDR < 0.1). Reduced expression of miR-205 together with greater expression of specific target mRNA genes (*PCSK1*, *SERPINA1, CARTPT)* in the HI calves suggested a relationship between these mRNA and miRNA. Furthermore, co-regulatory network analysis of the proteomic data revealed interactions between PCSK1, SERPINA1 and CARTPT, and proteins involved in cellular proliferation, metabolism and GnRH signalling, highlighting a role for these proteins in mediating the intersection between enhanced metabolic status with reproductive signalling in young bull calves.

## Introduction

The initiation of reproductive development in the male calf is a complex process mediated by the hypothalamic-pituitary–testicular (HPT) biochemical signalling axis. The hypothalamus is widely acknowledged as the homeostatic regulator of the body^1^ with metabolic cues produced in various tissues conveying the peripheral status of the body onto the hypothalamus, which in turn elicits subsequent downstream effects. These metabolic signals, including insulin-like growth factor-1 (IGF-1), insulin, leptin and adiponectin, can signal through neurons in the hypothalamus which can subsequently stimulate the secretion of gonadotropin releasing hormone (GnRH), a key gatekeeper of reproductive development. The anterior portion of the pituitary gland is the principal regulator for growth, metabolism and reproduction via the synthesis and subsequent secretion of an array of hormones that control these functions in multiple peripheral organs^2^. Following GnRH signalling within the anterior pituitary, the gonadotropins; follicle-stimulating hormone (FSH) and luteinising hormone (LH) are secreted^3^ which, in turn, signal to the testes, leading to synthesis of testosterone within the Leydig cells as well as contributing to spermatogenesis. Additionally the anterior pituitary is also responsible for the production and release of other hormones including those related to stress (adrenocorticotropic hormone), growth (growth hormone), fertility (prolactin) and metabolic rate (thyroid stimulating hormone).

Due to the role of peripheral metabolic cues towards signalling within the HPT axis, changes in dietary intake or metabolic status can significantly impact reproductive development. For example, nutritional restriction during the postnatal period can impact the timing of reproductive maturity by inhibiting GnRH release and therefore the release of LH^4,5^. Additionally, research has shown that malnutrition constitutes a greater stress to spermatogenesis in prepubertal males than in post pubertal males^6^, with restricted feed during calf-hood known to impede the hypothalamic GnRH pulse generator and impair steroidogenesis in the testes, delay puberty and decrease testicular weight at 70 weeks of age^7^. Conversely, studies have shown that a high plane of nutrition before 31 weeks of age results in earlier onset of puberty in Holstein–Friesian bulls^5,8^. Furthermore, enhanced metabolic status during the early calf-hood period has been shown to positively impact the hypothalamic GnRH pulse generator and its action on the anterior pituitary gland, thereby advancing the age of onset of puberty in bulls^6^.

A major feature of the gonadotropin profiles of developing bulls is a change in LH pulse frequency early in life^9^. In particular, studies have shown greater systemic concentrations of LH in bulls at 4 months of age compared to earlier and later ages when measured on a monthly basis up to 10 months of age^9^. Moreover, studies have shown that both the systemic concentrations of LH and frequency of episodic peaks of LH were greater in 4 month old bulls compared to 1 and 5 month old bulls^10,11^. The transient rise in anterior pituitary-derived systemic LH occurs between approximately 8–20 weeks of age in bull calves^12^ and when offered a high plane of nutrition during the first six months of life, bull calves have been shown to have an earlier and greater rise of LH^13^. Enhanced nutrition during this critical period can directly impact hypothalamic GnRH pulsatility, ultimately leading to enhanced LH pulsatility as well as testosterone synthesis and secretion^6,14^, which in turn, results in larger testes and puberty attainment at a younger age^5,6,14^. Thus, the reproductive axis has the capacity to respond to changing systemic concentrations of metabolic signals^15^ and it is clear that a high plane of nutrition in the early calf-hood period can advance puberty. Indeed, studies have shown large transcriptomic differences in the testes parenchyma of bull calves fed differentially up to 18 weeks of age^16^ and 24 weeks of age^17^. In each of these studies, enhanced dietary intake resulted in greater expression of genes involved in cholesterol biosynthetic processes as well as Sertoli cell maturation. Moreover, English et al.^18^ reported greater numbers of Sertoli cells as well as more mature spermatogenic cells in bull calves fed an enhanced plane of nutrition between 2–18 weeks of age. Furthermore, English et al.^16^ also reported differential expression of genes within the anterior pituitary of the same bull calves, highlighting differential expression of genes related to cell cycle processes. However whilst large differences were evident in the testes parenchyma work of English et al.^16^, much fewer differences were apparent within both the hypothalamus and anterior pituitary at 18 weeks of age. Thus the molecular mechanisms through which the effect of enhanced nutrition during early life on reproductive development through the HPT signalling axis is mediated are yet to be elucidated fully. The objective of this study was to evaluate the global transcriptomic (miRNA and mRNA) and proteomic response of the anterior pituitary in bull calves fed either a high (HI) or moderate (MOD) plane of nutrition during the first 12 weeks of life. A secondary objective was the integration of transcriptomic and proteomic data using co-regulatory network analyses, for the identification of key regulatory genes, which may hold potential for use in genomic selection processes. The ability to genomically select bulls that are better able to respond to enhanced early life nutrition is of benefit due to the industry goal of collecting semen from genetically elite and genomically selected young sires at an earlier age. This study builds on our earlier work^16,18^ and targets an earlier stage of development. Moreover whilst the current study is focused on the anterior pituitary, we have undertaken complementary analyses on the same calves in both the hypothalamus^19^ and the testes^20^.

## Methods

### Animal model and tissue collection

Tissue samples used in this study were derived from a larger study examining the effect of varied plane of nutrition during early life on the physiological and molecular control of reproductive development in the bull calf^21^, thus the animal model is only briefly described here. Holstein–Friesian bull calves, sourced from four high herd health status commercial dairy farms in Ireland, were blocked on age, bodyweight, sire (15 sires in total) and farm of origin (4 farms) and assigned to one of two dietary groups: a high (HI; n = 15) or a moderate (MOD; n = 15) plane of nutrition. Additionally, treatment groups were balanced for dam parity and blood ZST score, which reflected a proxy for immunoglobulin concentration in the blood, indicating colostrum derived passive immunity status^21^. From 2–12 weeks of age, calves received their respective dietary allowance to support target growth rates of > 1 kg/day and 0.50 kg/day for the HI and MOD groups, respectively. Specifically, calves in the HI group received 1,500 g of milk replacer (10 L/day; 4.89 Mcal/kg dry matter) with ad libitum concentrate (3.82 Mcal/kg dry matter), while MOD calves received 500 g of milk replacer (4 L/day; 4.89 Mcal/kg dry matter) with a maximum concentrate allowance of 0.5 kg/day (3.82 Mcal/kg dry matter). All calves across both treatment groups had continuous access to fresh water and approximately 0.5 kg of hay (3.84 Mcal/kg dry matter) daily. All calves were weighed at the beginning and end of the trial and also at weekly intervals. At 12 weeks of age all calves were euthanized through a lethal dose of sodium phenobarbital (1 mL/1.4 kg bodyweight) administered intravenously. The brain was subsequently removed and the pituitary gland was isolated from the sella turcica and weighed following which the anterior and posterior lobes were separated. All instruments used for tissue collection were sterilised and treated with RNAzap (Ambion, Dublin, Ireland) prior to use. Tissue samples were subsequently snap frozen in liquid nitrogen and stored at −80° C pending further processing. Care was taken at the time of sampling to ensure that anatomical zones or sections of the anterior pituitary of each bull were evenly represented across portions designated for subsequent proteomic and transcriptomic analyses.

### Global transcriptomic analyses

RNA isolation and RNA-sequencing are described in full in Coen et al.^20^. Briefly, the Qiagen RNeasy Plus Universal Mini Kit was used to isolate total RNA from all anterior pituitary samples (n = 30), according to the manufacturer’s instructions including adherence to appendix C for the isolation of small RNAs. An average of 90 mg of tissue was used for isolation of total RNA. RNA yield and quality were assessed by measuring the absorbance at 260 nm with a Nanodrop spectrophotometer and determining the RNA integrity number (RIN) using the RNA 6000 Nano Lab Chip kit on the Agilent 2100 Bioanalyser. All samples yielded RIN values of 8 or higher and were of suitable quality for RNA-sequencing. mRNA-seq and miRNA-seq library preparation and sequencing were undertaken by a commercial sequencing facility (Macrogen Europe Inc., Amsterdam, The Netherlands). Individual cDNA libraries were prepared from all 30 total RNA samples using the Illumina Truseq stranded mRNA kit for mRNA-seq and the Illumina small RNA kit for miRNA, according to the manufacturer’s instructions. mRNA-sequencing and miRNA-sequencing were undertaken on an Illumina NovaSeq (150 bp paired end sequencing) and an Illumina HiSeq 2500 sequencer (50 bp single end sequencing), respectively.

Full details related to RNA-sequencing analysis are described in full in Coen et al.^20^and are only briefly outlined here. Raw sequencing reads from both mRNA- and miRNA-seq experiments were firstly assessed for sequencing quality using FastQC (version 0.11.8^22^;). Cutadapt software (version 1.18.8^23^;) was used to remove Illumina sequencing indexing adapters from all reads. Cutadapt was also used to remove short (shorter than 15 bp) and long (longer than 28 bp) reads from the miRNA dataset. Retained reads from the miRNA dataset were subsequently filtered for other species of bovine short RNA (rRNA, tRNA, snRNA, snoRNA, downloaded from https://rnacentral.org/). The miRDeep2 package (version 2.00.8) together with the bovine reference genome (ARS-UCD1.2) and the known bovine mature miRNA sequences and their precursor sequences from the miRBase database were used to profile miRNA expression. Bowtie (version 1.1.1) and STAR (version 2.5.2.b^24^;) were used to align miRNA reads and mRNA reads, respectively to the bovine reference genome (ARS-UCD1.2). Aligned reads were subsequently quantified using miRDeep2 and STAR for miRNA and mRNA datasets, respectively. The R (v2.14.1) Bioconductor package, edgeR (version 3.26.7) was then used to determine miRNA and mRNA genes differentially expressed between HI and MOD calves. Differentially expressed genes were defined as those with a Benjamini–Hochberg false discovery rate (FDR) of less than 10% and a fold change value greater than 1.5. Target mRNA genes of differentially expressed miRNA were subsequently predicted using TargetScan (release 7.2; http://www.targetscan.org/vert_72/).

### Global proteomics analysis

A detailed description of the sample preparation and mass spectrometry analysis is provided in full in Coen et al.^20^. Proteins were extracted from pituitary tissue sample (average tissue weight of 80 mg per sample) using a tissue homogenizer (TissueLyser II, QIAGEN) and digested using a commercial iST Kit (PreOmics, Germany). Mass spectrometry analysis was subsequently undertaken on a Q Exactive HF-X mass spectrometer. Resultant raw mass spectrometry data were then processed using MaxQuant (version 1.6.2.3), followed by protein identification using the integrated Andromeda search engine^25^. The R package SRMService^26^ was used to normalise the data and subsequently compute differences in protein abundance between HI and MOD treatment groups.

### Integration of miRNA, mRNA and proteomic results

In order to evaluate the relationship between miRNA, mRNA and proteomic datasets an integrative analysis was conducted whereby differentially expressed miRNAs and their target mRNAs that were also differentially expressed were evaluated for their interactions within the proteomics data. A weighted co-expression network analysis (WGCNA) was then undertaken on the proteomics data and resultant networks mined for genes (which were targeted by differentially expressed miRNA) using the WGCNA R software program^27^. Label-free quantitation intensity values were firstly Log_2_ transformed in R. The automatic network construction and module detection method was subsequently used in WGCNA to generate unsigned co-expressed networks. Adjacency matrices of proteomics data were then calculated to reach scale-free topology of the network (R^2^ > 0.09) for the dataset by raising the co-expression matrix to a soft-threshold power of 15. The topology overlap matrix was then calculated, followed by application of average linkage hierarchical clustering to the topology overlap matrix resulting in the grouping of modules of co-expressed proteins. Modules or clusters of co-expressed proteins were then mined for genes of interest based on the relationship between differentially expressed miRNA and predicted target mRNA genes which were also differentially expressed.

### Biological pathways analysis

Ingenuity Pathway Analysis (IPA; Qiagen Inc., https://www.qiagenbioinformatics.com/products/ingenuitypathway-analysis^28^) was used to determine biological pathways and functions enriched based on miRNA, mRNA and proteomic analyses of anterior pituitary of bull calves fed either a HI or MOD diet during early life.

## Results

### Animal performance

Full details related to animal performance during the trial including dietary intake, growth rates, hormone and metabolite profiles are presented in Coen et al.^21^. Briefly, over the course of the 10-week differential feeding trial HI calves consumed more milk replacer compared to MOD calves (P < 0.0001), resulting in greater growth rates for the HI calves (HI: 0.88 kg/d; MOD: 0.58 kg/d; P < 0.001). At euthanasia at 12 weeks of age, HI calves were 24.7% heavier than MOD calves (HI: 112.4 kg; MOD: 87.7 kg; P < 0.001). Pituitary weight was greater in HI calves compared to MOD (HI: 0.68 g; MOD: 0.54 g; P < 0.01).

### miRNA-seq analysis

miRNA sequencing resulted in the generation of an average of 14 million reads per sample, with an associated alignment rate of 92.5% on average. Five miRNA were identified as differentially expressed between HI and MOD dietary treatment groups (FDR < 0.1; fold change > 1.5) (Table 1). Additionally, TargetScan analysis revealed predicted target mRNAs of the differentially expressed miRNA (Supplementary Table 1). Biochemical pathway analysis of target mRNA genes revealed significant enrichment (P < 0.05) of pathways including insulin receptor signalling, oxidative phosphorylation, GnRH signalling and both AMPK and sirtuin signalling. Details of all enriched pathways pertaining to mRNA targets of differentially expressed miRNA are outlined in full in Supplementary Table 2. Raw sequencing reads and gene counts for each sample utilised in this study have been deposited within NCBI’s Gene Expression Omnibus and are available through GEO ID GSE277616.

**Table 1:** Differentially expressed miRNA in the anterior pituitary of bull calves fed either a high or moderate plane of nutrition between 2–12 weeks of age.

miRNA	logFC	FDR
bta-miR-205	−0.65402	0.029068
bta-miR-451	0.660517	0.05134
bta-miR-144	0.672548	0.037952
bta-miR-10172-3p	0.916787	0.003388
bta-miR-2419-3p	1.606147	0.05134

### mRNA-seq analysis

**Fig. 1:**
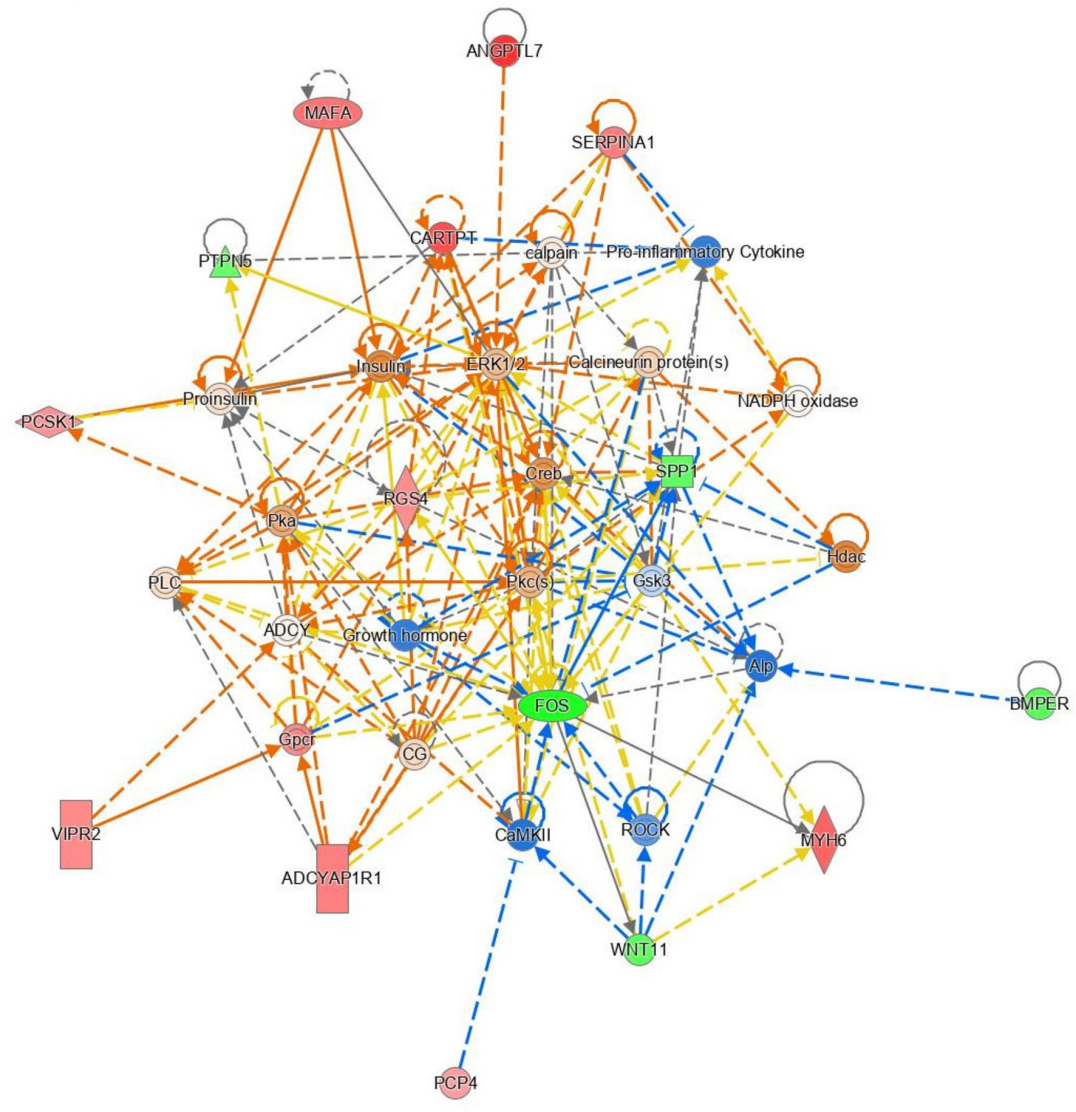
Network of genes involved in endocrine system development and function altered in the anterior pituitary of bull calves offered high or moderate planes of nutrition for the first 12 weeks of life. The network (network 1) is displayed graphically as nodes (genes). The node colour intensity indicates the expression of genes, with green representing down-regulation in calves fed the high plane of nutrition compared to those fed a moderate plane of nutrition up to 12 weeks of age. The network image was generated through the use of Ingenuity Pathway Analysis (QIAGEN Inc., https://www.qiagenbio-informatics.com/products/ingenuity-pathway-analysis)^21^.

An average of 71 million sequencing reads were generated through mRNA sequencing across all 30 samples. Alignment of trimmed sequencing reads to the bovine genome resulted in an average mapping rate of 87% across all samples. Following the removal of lowly expressed genes within edgeR, a total of 12,310 genes remained for differential expression analysis. EdgeR analysis resulted in the identification of 37 differentially expressed genes (FDR < 0.1; fold change > 1.5) between treatment groups. Differentially expressed genes and their fold changes are presented in Table 2. Of particular interest were *PCSK1*, *SERPINA1* and *CARTPT*, which through TargetScan analysis were identified as predicted mRNA targets of the differentially expressed miRNA, miR-205. Pathway analysis of differentially expressed genes revealed significant enrichment (P < 0.05) of biochemical pathways including those related to intracellular signalling such as G-protein coupled receptor signalling, tight junction signalling and cAMP-mediated signalling (Supplemental Table 3). Network analysis through IPA also revealed a network (Network 1, Supplemental Table 4) of interest related to endocrine system development and function, which is presented in Fig. 1. Additionally, biological functions affected included those related to endocrine system development and cellular proliferation (Supplemental Table 5), enriched endocrine functions are presented in Table 3. Raw sequencing reads and gene counts for each sample utilised in this study have been deposited within NCBI’s Gene Expression Omnibus and are available through GEO ID GSE278369.

**Table 2:** Differentially expressed mRNA in the anterior pituitary of bull calves fed either a high or moderate plane of nutrition between 2–12 weeks of age.

Gene ID	HGNC	logFC	FDR
ENSBTAG00000020247	*ADCYAP1R1*	1.077204	0.057934
ENSBTAG00000015340	*ANGPTL7*	1.769053	0.018859
ENSBTAG00000016227	*ASCL1*	0.687413	0.037683
ENSBTAG00000017716	*BEGAIN*	0.817097	0.066937
ENSBTAG00000010866	*BMPER*	−0.82555	0.056563
ENSBTAG00000050066	*C15H11orf87*	−0.58627	0.014882
ENSBTAG00000047486	*CARTPT*	1.537872	0.050582
ENSBTAG00000010793	*CCDC80*	0.626261	0.031957
ENSBTAG00000044073	*CD248*	0.675041	0.086867
ENSBTAG00000021487	*CIART*	−0.8022	0.012698
ENSBTAG00000038854	*CLEC2L*	0.825923	0.091294
ENSBTAG00000005784	*CSMD2*	0.775683	0.066937
ENSBTAG00000002110	*DMGDH*	0.731489	0.025773
ENSBTAG00000051577	*FAM163B*	0.920355	0.014732
ENSBTAG00000004322	*FOS*	−1.23949	0.060747
ENSBTAG00000018948	*HIF3 A*	−0.92856	7.87E-04
ENSBTAG00000011377	*KCNMB2*	−1.19188	2.17E-04
ENSBTAG00000026344	*MAFA*	1.207624	0.064364
ENSBTAG00000012252	*MOCOS*	0.606357	0.060179
ENSBTAG00000040053	*MYH6*	1.3613	2.66E-04
ENSBTAG00000010277	*NKX2-2*	−0.68884	0.037683
ENSBTAG00000008940	*NPTX1*	0.814389	0.091294
ENSBTAG00000012630	*PAMR1*	0.674305	2.66E-04
ENSBTAG00000051421	*PCP4*	0.673546	0.036857
ENSBTAG00000020843	*PCSK1*	0.830524	0.031957
ENSBTAG00000020257	*PTPN5*	−0.67161	0.012945
ENSBTAG00000046277	*RGS4*	0.908697	0.032415
ENSBTAG00000018843	*SERPINA1*	1.111508	0.024524
ENSBTAG00000051461	*SERTM1*	−0.61811	0.037683
ENSBTAG00000005260	*SPP1*	−0.74628	2.17E-04
ENSBTAG00000020060	*TXNIP*	−0.62066	0.003102
ENSBTAG00000037649	*VIPR2*	0.949573	0.040099
ENSBTAG00000006232	*WDR86*	0.869414	0.032415
ENSBTAG00000049788	*WFDC1*	0.972585	0.056563
ENSBTAG00000010820	*WNT11*	−0.78139	0.064364
ENSBTAG00000048515		−1.02974	0.034944
ENSBTAG00000051317		1.156499	0.014882

**Table 3:** Endocrine functions enriched based on differentially expressed mRNA in the anterior pituitary of 12-week-old bull calves fed either a high or moderate plane of nutrition.

Diseases or functions annotation	p-value	Molecules
Glucose tolerance	1.67E-06	*ADCYAP1R1,CARTPT,CCDC80,MAFA,PCSK1,SPP1,TXNIP*
Quantity of insulin in blood	3.28E-05	*ADCYAP1R1,CARTPT,CCDC80,PCSK1,SPP1*
Differentiation of neuroendocrine cells	3.88E-05	*ASCL1,NKX2-2,WNT11*
Secretion of hormone	0.000142	*CARTPT,MAFA,NKX2-2,RGS4*
Concentration of hormone	0.000147	*ADCYAP1R1,CARTPT,CCDC80,PCSK1,SPP1,VIPR2*
Depolarization of lutenizing hormone-releasing hormone neurons	0.00143	*CARTPT*
Dispersion of endocrine cell lines	0.00286	*SPP1*
Development of chromaffin cells	0.00286	*ASCL1*
Regulation of endocrine system	0.00286	*CARTPT*
Differentiation of chromaffin cells	0.00286	*ASCL1*
Insulin sensitivity	0.00476	*SPP1,TXNIP,VIPR2*
Catabolism of androgen	0.00713	*SPP1*

### Global proteomic analysis

Protein quantification and identification, undertaken through MaxQuant software (v1.6.2.3), resulted in the identification of 4,132 proteins across the anterior pituitary samples examined. The total number of proteins represents those with at least two peptides and a maximum of 10 missing values per protein. However despite the identification of 4,132 proteins, no protein passed multiple testing correction to be deemed statistically significantly different between the two dietary treatment groups (adj. P-value 0.1; fold change > 1.5). The full list of proteins identified in the anterior pituitary tissue and their associated uncorrected p-values are presented in Supplemental Table 6. Proteomics data generated in this analysis have been uploaded to the ProteomeXchange Consortium via the PRIDE (http://www.ebi.ac.uk/pride) partner repository^29^ with the data identifier PXD055940.

### Integrative network analyses

**Fig. 2:**
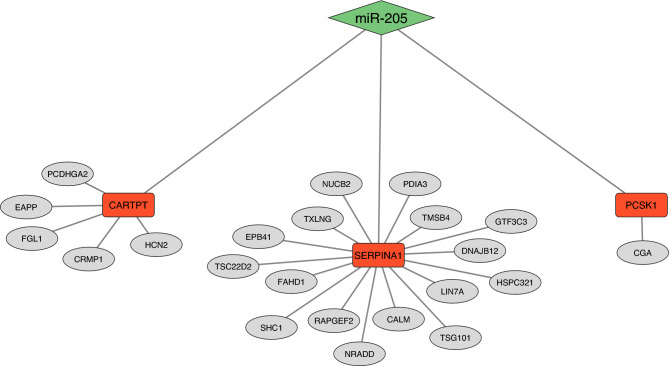
Interaction between miR-250, *CARTPT*, *PCSK1* and *SERPINA1* across miRNA, mRNA and proteomic datasets. miR-250 (green node) was down-regulated in the HI calves, whilst predicted target genes of miR-250 (*PCSK1*, *CARTPT* and *SERPINA1*) were up-regulated (red nodes) in the same group, indicated a direct relationship between these genes. Proteins interacting with PCSK1, CARTPT and SERPINA1 derived from the co-regulatory network analysis are also presented (grey nodes).

Co-regulatory network analysis of proteomics data resulted in the formation of 12 separate networks of proteins. Correlated, co-regulated proteins within each network are detailed in full in Supplementary Table 7. As mentioned above, one miRNA, miR-205 as well as three of its corresponding target mRNA genes, *PCSK1*, *SERPINA1*, and *CARTPT* were differentially expressed between the HI and MOD dietary groups, suggesting a direct relationship between the miRNA and predicted target mRNA genes. Moreover, through the network analysis of the proteomics dataset, PCSK1, SERPINA1, and CARTPT were identified as hub proteins across three separate networks of co-regulated proteins. Proteins directly interacting with PCSK1, SERPINA1 and CARTPT are presented in Fig. 2.

## Discussion

Enhanced metabolic status during the early life period is known to elicit earlier reproductive development and consequently earlier sexual maturation in bull calves^5,14^. This effect is mediated though metabolic cues from peripheral tissues signalling to the hypothalamus, which in turn stimulate synthesis and secretion of GnRH, gonadotropin release and ultimately eliciting the final downstream effects on the testes. Indeed, enhanced early life nutrition in the calves used in the current study resulted in earlier development of the testes as evidenced by greater weight of the testes in the HI calves^20^, and this effect has been replicated in other studies conducted using independent populations of bull calves^16–18^. However, although indices of advanced reproductive development were apparent in the aforementioned studies, evidence for an effect of enhanced nutrition on the molecular regulation of the anterior pituitary gland function has been limited. For example, differential expression of genes that are key drivers of this effect within the pituitary including for example FSH, LH, and GnRH receptor (GnRHR) was not observed in either the current study nor that of English et al.^16,18^. Despite this, differential feeding in the current study resulted in the enrichment of the G-protein coupled receptor (GPCR) signalling pathway in the anterior pituitary. The GnRH receptor belongs to the GPCR family of signalling molecules^30,31^ which regulate a diverse array of physiological functions and ligands, but equally are highly conserved in their common function of transducing an extracellular signal across a biological membrane via a change in receptor protein conformation^32,33^. The enrichment of the GPCR signalling pathway in the current study is a consequence of the differential expression of protein coding genes including *ADCYAP1R1, FOS, RGS4 and VIPR2*, all of which were up-regulated in the HI calves, with the exception of *FOS* which displayed greater expression in the MOD calves. The up-regulation of *ADCYAP1R1, RGS4* and *VIPR2* suggests an overall up-regulation of the GPCR signalling pathway in the HI calves, apparent through the specific functions of each of these genes. For example, *ADCYAP1R1* encodes a type 1 adenylate cyclase activating polypeptide receptor, *RGS4* functions as a regulator of G protein signalling and *VIPR2* encodes a G protein receptor. Indeed differential expression of *ADCYAP1R1* was of particular interest as this protein has previously been shown to regulate the release of various pituitary-derived hormones. For example PACAP, the protein encoded by *ADCYAP1R1* can lead to increased mRNA expression of the β subunit of LH (LHB) as well as the α subunit of LHB (*CGA* gene) as well as regulating the transcription of FSHB and GnRHR^34^. Overall the differential expression of the aforementioned protein-coding genes suggests a greater requirement for GPCR signalling in the HI calves and, when combined with the greater expression of *ADCYAP1R1*, suggests greater potential for transcription of gonadotropins in the HI calves, which was evident from the systemic concentrations of these hormones in the calves used in this study^21^. However it must be noted that our analysis was conducted on tissue samples and not on individual cell types within the anterior pituitary, thus it is not possible to determine if the differential expression of genes within the GPCR signalling pathway were specifically related to gonadotrophs within the anterior pituitary, and may be attributed to other cell types within the anterior pituitary including for example somatotrophs, which comprise the largest cell population within the anterior pituitary as well as thyrotrophs or corticotrophs.

In addition to the aforementioned effect of plane of nutrition on genes involved in GPCR signalling, differential expression of protein coding genes involved in endocrine functions was also apparent in the pituitary between the HI and MOD calves. This was evident through the enrichment of biological functions associated with endocrine system development and function (Table 3) as well as through network 1 (Fig. 1) which included genes involved in endocrine system development, function and organ morphology. Indeed, Fig. 1 also contains differentially expressed genes of the G protein-coupled receptor signalling pathway further establishing a role for these genes towards endocrine or hormone processing within the anterior pituitary. Interestingly, Fig. 1 also includes the hormones insulin and growth hormone (GH). Growth hormone is known to elicit its anabolic effect through stimulation of insulin as well as IGF-1. Thus, the inclusion of both insulin and GH within Fig. 1 may indicate a contribution of insulin towards growth of the pituitary gland as a consequence of enhanced early life dietary intake. Although GH was not measured in the calves used in this study, systemic concentrations of insulin, glucose and IGF-1 were all greater in the HI calves^21^. Indeed pathway analysis of the differentially expressed protein coding genes highlighted enrichment of the cellular growth and proliferation function between calves fed different planes of nutrition. Moreover, the pituitary gland was heavier (on an absolute basis) in the HI calves used in the current study; thus insulin and GH/IGF-1 may be contributing to this growth. Indeed both insulin and IGF-1 have been shown to act directly on the HPT signalling axis, influencing reproductive development. For example, temporal secretion patterns of IGF-1 and LH were reported in Holstein–Friesian bulls, highlighting the role of IGF-1 in the regulation of the early gonadotropin rise and subsequent reproductive development^5^, whilst treatment with IGF-1 has been shown to increase LH secretion in castrated rams^35^. Additionally, Dance et al.^5^ observed a relationship between the combination of IGF-1 and FSH on proliferation of Sertoli cells cultured in vitro from testicular tissue of 8-week old calves. Furthermore, insulin has been shown to impact reproductive signalling. For example in a study conducted on rams, Blanche et al.^36^ reported a relationship between improved nutrition and subsequent increased circulating insulin concentrations with increased GnRH and LH secretion. Moreover, using mice developed with a neuron-specific disruption in the insulin receptor gene, Bruning et al.^37^ reported a 60% reduction in plasma concentrations of LH and impaired spermatogenesis as well as a reduction in the number of Leydig cells within the testes, when compared to wild type mice, highlighting the influence of insulin on reproductive signalling and development across the pituitary and testes tissues. However as stated above, our analyses were conducted on tissue samples that contain a heterogeneous complement of cell types, thus it is not possible to attribute differences related to growth processes as directly derived from somatotropic related processes. In the current study the role of insulin towards mediating earlier reproductive development is, however, further supported through the identification of insulin as an upstream regulator of the *ADCYAP1R1* gene, which as mentioned above has functions in the transcription of the gonadotropins. Furthermore, the importance of insulin to earlier gonadotropin secretion within the anterior pituitary^6,7,38^ is further apparent through the biological functions associated with endocrine system development and function (Table 3), which included those related to glucose tolerance and quantity of insulin in blood, as well as other functions related to hormone secretion and concentration of hormones in general. Across each of these functions, two genes were of particular interest, namely *CARTPT* and *PCSK1*, both of which were up-regulated in the HI calves. *CARTPT* encodes a preprotein that is proteolytically processed to generate multiple biologically active peptides, with the peptides involved in diverse physiological functions including appetite, energy balance, maintenance of body weight and the stress response. *CARTPT* is a satiety factor closely associated with the actions of leptin and NPY; this anorectic peptide inhibits both normal and starvation-induced feeding and completely blocks the appetite response induced by NPY and regulated by leptin in the hypothalamus. Similarly, in a contemporary study undertaken in heifer calves offered similar contrasting dietary regimens up to 22 weeks of age, greater expression of genes related to satiety was evident within the hypothalamus of heifers fed the higher plane of nutrition^39^. Moreover, *CARTPT* was identified within an anterior pituitary gene co-expression network, which was positively correlated with indices of earlier reproductive development in bull calves including stage of spermatogenesis and systemic concentrations of LH^40^, implicating this gene in reproductive development in early life. *PCSK1* encodes a member of the subtilisen-like proprotein convertase family which includes proteases that process protein and peptide precursors trafficking through a secretory pathway. Specifically, *PCSK1* is involved in the processing of hormones and other protein precursors with relevant substrates including the appetite suppressing proopiomelanocortin (POMC), somatostatin, insulin and agouti-related protein (AGRP), which function in the stimulation of appetite. Similarly, in a study evaluating the effect of enhanced early life nutrition in bull calves up to 18 weeks of age^16^, *PCSK1* was also upregulated in the anterior pituitary of calves offered the higher plane of nutrition. Moreover, *PCSK1* was included within a gene co-expression network which was positively associated with systemic concentrations of both testosterone and LH as well as with markers of testicular development (stage of spermatogenesis, Sertoli cell number)^40^. Taken together, these findings indicate an enhanced endocrine function within the HI calves, with higher insulin concentrations potentially driving this effect. Moreover, results indicate satiety within the HI calves compared to the MOD fed calves, which may facilitate a precocious commencement of reproductive development neuroendocrine signalling as a consequence of enhanced metabolic status in early life.

The differential expression of some of the aforementioned genes may have been a direct effect of the differential expression of miRNA between HI and MOD calves in the current study. For example miR-144 was up-regulated in HI calves with corresponding target mRNA genes (*FOS*, *KCNMB2*, *PTPN5*) all down-regulated in the same calves. Similarly, greater expression of miR-2419-3p and lower expression of miR-205 in the HI calves coincided with differential expression of target genes including lower expression of *HIF3A* and greater expression of *CARTPT*, *PCSK1* and *SERPINA1*, respectively. The differential direction of effect between the differentially expressed miRNA and their target genes, which were also differentially expressed, indicates a potential relationship between the miRNA and their target mRNA. Whilst some of the miRNA identified as differentially expressed between the HI and MOD groups have previously been implicated in other effects in cattle, the current study is the first report of miRNAs related to the intersection between metabolic status and reproductive development as a consequence of enhanced early life nutrition. For example, evidence for an involvement of the differentially expressed miRNAs and metabolism is established through the differential expression of miR-205, which has been shown to have important functions towards adipogenesis and lipid metabolism in hepatic tissue^41^ and miR-2419-3p which was previously reported as associated with fatty acid profiles in muscle^42^ as well as regulating fatty acid composition in Nellore cattle^43^. Additionally, miR-2419-3p was also up-regulated in the arcuate nucleus region of the hypothalamus in the HI calves used in the current study^19^, highlighting a function for this miRNA based on prevailing dietary intake. Moreover, miR-205 and miR-451 were both differentially expressed in follicular fluid of cows divergent for post-calving metabolic status^44^, highlighting a possible role for these miRNA as intermediaries in the interplay between metabolic homeostasis and reproductive function. Furthermore, a role for the differentially expressed miRNAs in the current study towards reproductive development and function has previously been established. For example, Ye et al.^45^ observed miR-451 to be up-regulated in pituitary cells in pigs following treatment with GnRH. Moreover, Gao et al.^46^ observed differential expression of miR-144 between neonatal and mature bovine testes, highlighting a possible role for this miRNA in testicular development. Indeed, the varying roles of the differentially expressed miRNA reported in the current study are further established through the diverse pathways enriched based on the target genes for each miRNA outlined in Supplementary Table 3. Overall, miRNA differentially expressed between HI and MOD dietary groups suggest a role towards mediating the intersection between metabolic status and subsequent reproductive development, with miRNAs potentially controlling the differential expression of protein coding genes identified within the pituitary also.

Despite identifying over four thousand proteins within the anterior pituitary tissue following global proteomics analysis, none were identified as significantly differentially abundant between the HI and MOD dietary groups once correction of p-values for multiple testing was applied. Notwithstanding this outcome, a secondary objective of this study was to evaluate the interaction amongst proteins identified within the anterior pituitary. Of particular interest were proteins whose mRNA expression may have been affected by corresponding miRNA expression. This resulted in a focus on the target genes of miR-205 including *SERPINA1*, *PCSK1* and *CARTPT*, which encode proteins identified as hub proteins within three separate networks of co-regulated proteins following co-regulatory network analysis of the anterior pituitary proteome. Whilst the functions of both PCSK1 and CARTPT have been described above, SERPINA1 is a serine protease inhibitor belonging to the serpin superfamily of protease inhibitors. While each of these proteins were identified as hub proteins in their respective networks, their potential impact varied with differences in the number of interactions for each protein. Specifically, CARTPT was identified as regulating five other proteins, whilst SERPINA1 was regulating sixteen proteins while at the same time also being regulated by a large number of other proteins, with PCSK1 regulating only one other protein. CARTPT, the protein previously described in relation to satiety was co-regulated with proteins involved in neurodevelopment and the establishment of neuronal connections within the brain, including CRMP1 and PCDHGA2. Moreover, CARTPT was also regulating the abundance of EAPP, a protein involved in cellular proliferation, suggesting that the increased dietary intake above maintenance nutrient requirements in the HI calves may have resulted in a state of metabolic satiety allowing the energy to be diverted towards brain tissue development and growth. Interestingly, CARTPT was also co-regulated with HCN2, which is a potassium and sodium ion channel, involved in GnRH secretion from the hypothalamus through hyperpolarisation-activated currents within GnRH neurons. Furthermore, in addition to its role in GnRH secretion, studies have shown a potential role for such ion channels, including HCN2, towards rhythmic hyperpolarisations of pituitary gonadotropes^47,48^, potentially indicating a role for CARTPT and HCN2 in modulating the effect of enhanced metabolic status with earlier reproductive development. Additionally, SERPINA1 may also represent an intersection between metabolic status with earlier reproductive development. This was apparent through the enrichment of pathways from the interactions of SERPINA1, including those related to metabolism, namely sirtuin and AMPK signalling pathways as well as those involved in hormone processing and reproductive signalling (synaptogenesis signalling, GnRH signalling and opioid signalling). Interestingly, SERPINA1 was co-regulated with proteins that have previously been implicated in pubertal development in cattle. Specifically, SERPINA1 was regulating TSG101, whilst SCG3 was regulating SERPINA1. Indeed, Dias et al.^49^ reported SNPs within both of these genes as being associated with puberty attainment in cattle. Furthermore, an evaluation of anterior pituitary gene co-expression networks in a contemporary study undertaken with bull calves up to 18 weeks of age reported a network containing *SCG3* as significantly positively correlated with greater systemic concentrations of testosterone and LH, as well as markers of testicular development including Sertoli cell number and stage of spermatogenesis^40^, highlighting the potential importance of SCG3 towards earlier reproductive development as a consequence of enhanced early life nutrition. Finally, although PCSK1 was identified as regulating only one other protein, the specific protein, CGA, is the α subunit for the gonadotropes, FSH, LH as well as for thyroid stimulating hormone. Thus, given the role of PCSK1 towards the processing of hormones, the interaction between PCSK1 and CGA is of interest in relation to the effect of enhanced early life nutrition on earlier reproductive development in bull calves. Moreover, in a contemporary study undertaken in heifer calves fed differentially up to 22 weeks of age, *CGA* expression in the arcuate nucleus region of the hypothalamus was up-regulated in calves fed a high plane of nutrition compared to a moderately fed group^39^, highlighting the role of CGA towards mediating metabolic status with earlier reproductive development.

## Conclusions

Results from this study show a clear effect of enhanced dietary intake up to 12 weeks of age on the transcriptome and proteome of the anterior pituitary gland. Differentially expressed protein coding genes between HI and MOD calves resulted in an enrichment of G-protein coupled receptor signalling, which is involved in GnRH receptor signalling, as well as an enrichment of processes related to neuroendocrine system development and function, highlighting a potentially important role for insulin in driving the differential gene expression profiles observed. Additionally, differentially expressed miRNA between HI and MOD dietary groups suggest a potential role towards mediating the intersection between metabolic status and subsequent reproductive development. Moreover, through the integration of miRNA and mRNA results, with proteomic data, an effect of the miR-205 miRNA on the expression of *PCSK1*, *SERPINA1* and *CARTPT* was established, with co-regulatory network analysis of the proteomic data further highlighting a role for these proteins in mediating enhanced metabolic status with earlier reproductive signalling. The data generated in this study broaden our knowledge in relation to potential molecular processes mediating the cross-talk between metabolic status and reproductive development. However, although the results generated in this study reveal interesting insights into the molecular response of the anterior pituitary gland to plane of nutrition during early life, the data generated is limited by the lack of information related to potential alterations to the proportion of various cells types within the anterior pituitary tissue. Specifically, it was not possible to infer whether proportions of cells including, gonadotrophs, thyrotrophs and somatotrophs, were impacted by augmented early life nutrition during early life, which may ultimately impact the propensity for production of specific hormones produced and secreted by these various cell types which may in turn impact development of other tissues or biochemical processes throughout the body, for example production of testosterone within the testes. Thus, further work is warranted in relation to uncovering any potential effect of enhanced early life nutrition on the population of the various cell types within the anterior pituitary gland. However, despite the aforementioned limitations, results generated in this study provide novel information of the underlying biology governing the anterior pituitary response to enhanced early life nutrition. Furthermore, these data contribute to the selection of robust biomarkers which may hold potential for the identification of bulls with superior genetic potential for earlier onset of puberty as a consequence of early life plane of nutrition.

## Supplementary Information

Supplementary Information.

## Data Availability

The sequencing data underlying this article are available in NCBI’s Gene Expression Omnibus at [https://www.ncbi.nlm.nih.gov/geo/] and is publicly available and can be accessed with unique GEO ID [GSE278369] and [GSE277616]. Proteomics data generated in this analysis have been uploaded to the ProteomeXchange Consortium via the PRIDE [http://www.ebi.ac.uk/pride] partner repository^22^ with the data identifier: PXD055940.
